# The feasibility and acceptability of an app-based cognitive strategy training programme for older people

**DOI:** 10.1186/s40814-023-01334-x

**Published:** 2023-06-30

**Authors:** Nikki Tulliani, Rosalind Bye, Michelle Bissett, Samantha Coutts, Karen P. Y. Liu

**Affiliations:** 1 School of Health Sciences, Western Sydney University, NSW Penrith, Australia; 2 Faculty of Health, Southern Cross University, QLD Gold Coast, Australia; 3 Translation Health Research Institute, Western Sydney University, Penrith, NSW Australia; 4 Department of Rehabilitation Sciences, The Hong Kong Polytechnic University, Hung Hom, Hong Kong

**Keywords:** Older people, Cognition, Perceptual encoding, Visual imagery, Intervention, Feasibility, Daily activities

## Abstract

**Background:**

Increasing numbers of people are living with mild cognitive impairment in later life and seeking therapy to maintain cognition to remain as independent as possible in daily life. Based on a review of the literature, an app-based programme using perceptual-encoding strategies called Enhancing Memory in Daily Life (E-MinD Life) was developed. An expert panel reviewed the programme’s appropriateness for older people with and without mild cognitive impairment. As part of the design process, the feasibility and acceptability of the E-MinD Life programme were then assessed in relation to its use by healthy older adults, with findings informing the application of the programme to older people with mild cognitive impairment in the future.

**Methods:**

Phase 1: The E-MinD Life programme was reviewed by an expert panel of occupational therapists. Experts rated the programme on a Likert scale and answered open-ended questions in relation to feasibility, clarity, and relevancy. Phase 2 involved field-testing the 9-week programme with a sample of nine healthy older people. Participants rated the acceptability of the programme on a Likert scale questionnaire. Data on recruitment rates and retention, and adherence and duration of sessions were collected to determine the feasibility of the programme. Responses to the Likert scale were analysed using descriptive statistics. Open-ended responses were categorised qualitatively using a constant comparative approach.

**Results:**

Phase 1: Experts indicated that the E-MinD Life programme was feasible and included relevant activities for community living. Although experts felt that an older user with mild NCD would be able to independently complete the programme, the qualitative analysis suggests formatting changes in future iterations of the programme to enhance visual clarity. Phase 2: All participants completed the 9-week programme. The average number of self-administered sessions attempted over the 9-week period was 13.44 (SD = 6.73) out of 18 scheduled sessions. Overall, most participants found the programme relevant, logical and easy to understand, and perceived it to be effective for functional cognitive problems.

**Conclusion:**

The E-MinD Life programme shows promise for inclusion into trial designs to determine the effectiveness of the cognitive strategy programme for older people with and without cognitive impairment.

**Trial registration:**

ClinicalTrials.gov, NCT03430401. Registered 1 February 2018.

**Supplementary Information:**

The online version contains supplementary material available at 10.1186/s40814-023-01334-x.

## Key messages regarding feasibility

- What uncertainties existed regarding the feasibility?
- The feasibility of recruiting enough participants to determine the effectiveness of the programme.
- The feasibility of older people correctly administering the programme and using the taught cognitive strategies.
- Future research is required to determine the E-MinD Life programme feasibility with older people with mild NCD.
- What are the key feasibility findings?
- It is feasible to recruit and retain older people to participate in a 9-week cognitive strategy programme administered via an iPad.
- Older people found the cognitive strategy programme acceptable and relevant and support further development and use of the programme.
- Occupational therapists had positive impressions of the programme.
- What are the implications of the feasibility findings for the design of the main study?
- The findings of this study provide important information about recruitment, adherence, and programme design (relevancy, clarity, effectiveness) of a cognitive strategy programme targeted at older people.
- Findings from this study indicate formatting changes to the programme are required prior to the main study to enhance useability.

## Background

As part of the normal ageing process, older people may experience deterioration in memory, which then affects their independence in daily activities [1]. The impact of declining memory on day-to-day activities is more pronounced for people with mild neurocognitive disorder (NCD) and major NCD. Implicit memory and explicit memory are types of long-term memory that support performance in day-to-day activities. Implicit memory (non-declarative memory) involves the unconscious and effortless recollection of previous experiences to aid in the performance of an activity [2, 3]. In comparison, explicit memory (declarative memory) requires conscious effort or intentional recall of specific experiences or information to aid activity performance [2]. Furthermore, memory encompasses three sequential stages: (1) encoding or acquisition of new information, (2) storage of information, and (3) retrieval of information [4]. Difficulties with encoding new information may contribute to age-related difficulties in episodic memory, a type of explicit memory, which stores details of past experiences [5]. Decreases in encoding efficiency occur as part of the normal ageing process. For older people, difficulties to encode information may be due to a reduced ability to independently initiate encoding strategies and/or adequately organise information so that learning can take place [6, 7]. Consequently, older people may benefit from interventions targeted at techniques to encode information.

During the early stages of cognitive decline, such as mild NCD and early-stage major NCD, the ability to perform basic activities of daily living (BADL) normally remains intact [8]. BADL include self-care activities such as bathing, toileting, dressing, or eating [9]. However, early stages of cognitive decline are often associated with a noticeable decline in the performance of instrumental activities of daily living (IADL) such as completing household chores, cooking, shopping, and managing finances [10–13]. Difficulties with completing IADL may impact a person’s ability to independently live in the community [14]. Therefore, effective interventions to maintain or improve IADL performance in people with mild NCD and early-stage major NCD may aid community living, reduce strain on carers, and limit or delay the need for assistance with IADL and therefore the need for home care services. Furthermore, such interventions may also be beneficial for healthy older people to partake. By participating in such interventions, healthy older people at risk of cognitive decline may delay the onset of cognitive decline or at a minimum increase the likelihood of them being able to experience a lesser decline in the capacity to perform IADL.

Occupational therapists assist older adults to maintain or improve their performance of IADL essential for independent community living. Occupational therapists consider transportation, shopping, telephone use, and meal preparation as important activities for older adults to remain living independently in the community [15]. Therapists need to know, and apply, the best approaches which can be used to assist older people to improve IADL when they experience memory decline.

### Memory interventions for improved daily life

Cognitive training and cognitive rehabilitation are non-pharmacological interventions aimed at promoting the maintenance of memory and functional cognition [16, 17]. These approaches are commonly used in occupational therapy practice. Cognitive training targets specific domains of cognition, such as memory, attention, and problem-solving. Examples of cognitive training include training in applied memory strategies and mnemonic techniques such as cueing, method of loci, and repeated attention and memory tasks [16]. In comparison, cognitive rehabilitation focuses on teaching a person strategies for learning new information or compensatory strategies to assist in activity performance specific to the individual’s needs and daily activities [16]. Examples of cognitive rehabilitation include memory retrieval techniques, activity or environment modification, and errorless learning [16, 18]. Cognitive training and cognitive rehabilitation are two intervention approaches that may assist with maintaining cognition at an optimal level during the ageing process.

Cognitive rehabilitation tends to work on the retrieval stage of memory such as the use of external aids such as notes, pill boxes, diaries, checklist, and alarms to assist with memory retrieval and remind an individual to complete a task [19]. This is a widely used approach in occupational therapy, using adaptation or compensatory strategies [20], whereas internally focussed strategies, such as those used in cognitive training, aim to improve or regain memory function. Occupational therapists use strategies such as repeated practice, mental imagery, and rehearsal that target the encoding and/or retrieval stages of memory [19].

It is important to understand specific aspects of these processes so that any planned intervention is informed by what is known about memory encoding and retrieval. This study specifically focused on developing an intervention that targeted perceptual-based memory encoding as there is evidence regarding the success of this approach with people with mild NCD [21]. Perceptual-based memory encoding involves converting visual information to memory, to assist with recall [22]. Fundamental to this approach are the processes of priming, including visual imagery and the ‘method of loci’ strategies.

Priming is a category of implicit or non-declarative memory in which exposure to a stimulus influences a person’s response to a succeeding stimulus, without conscious awareness, effort, or intention [23, 24]. A person may show improved memory when they have been subconsciously prepared and exposed to stimulus related to the activity [2, 23]. This improved performance is known as the priming effect [2].

Visual imagery and the ‘method of loci’ encode steps of tasks based on visual details [25]. Visual imagery is a form of sensory imagination [26] where associations are made between verbal and visual information and images are created in the mind to facilitate the learning and recall of information [27]. For example, a person might close their eyes and visualise themselves or another person performing an activity after watching a video of the activity being performed (perceptual priming), read the steps of the activity (semantic priming), or receive auditory information about the steps and context of the activity (auditory priming). The method of loci is a strategy that uses visual imagery [28, 29], whereby a person imagines themselves ‘placing’ items to be remembered in a familiar location. To retrieve and recall these items, they re-imagine themselves in the location recovering the items from where they were placed [29, 30].

The use of visual imagery has been shown to enhance attention and sequential processing of functional task performance for people with stroke [31, 32] and has been used as a memory aid technique for individuals following traumatic brain injury [25]. Studies using visual imagery and the method of loci memory training have demonstrated memory improvements associated with increased activation in parietal-occipital areas, responsible for visual perception and processing [33], as well as change in the neurochemistry in the hippocampus, responsible for learning and memory [34]. Visual imagery and the method of loci have also been shown to assist with age-associated memory decline in healthy older people [35, 36]. While explicit memory is impaired in people with mild NCD, their implicit memory and perceptual priming are typically preserved [24], and therefore, perceptual-based memory encoding strategies may assist older people with mild NCD to maintain IADL performance.

### Enhancing memory interventions through the use of technology

Increasing numbers of older people now use electronic devices such as smartphones and tablet computers to engage in cognitive stimulating activities and socialisation or to record, track, and communicate information about their health status [37]. Therefore, technological advances now make it possible to administer perceptual-based cognitive training at home on mobile and touchscreen devices such as touchscreen or tablet computers. Although the technology industry has developed and commercialised numerous ‘brain training’ computerised programmes claiming to improve memory and other cognitive skills, many are unregulated and lack evidence supporting their effectiveness [38]. However, several studies have emerged demonstrating that computerised cognitive interventions may improve cognition in older people with mild NCD [39–42].

Cognitive training and memory encoding require frequent and repetitive practice [43]. The convenience of being able to self-administer cognitive interventions at home may support these requirements. With increased episodes of intervention, the goal of strengthening cognitive processes, and subsequent IADL performance more generally, could be achieved.

Delaying or preventing cognitive decline is key, as mild NCD is a transitional period between normal ageing and a diagnosis of major NCD [43–47], with one study (*n* = 133) finding a conversion rate from mild NCD to Alzheimer’s type major NCD to be a 30.5% over an 18-month period [48]. As partaking in cognitive activities is correlated with a delay in the onset of memory decline in those who develop major NCD [49], designing effective interventions to support the preservation of memory and performance of IADL is vital.

## Methods

### Purpose of the study

This study sought to examine the feasibility of an app-based cognitive strategy programme using perceptual memory-encoding strategies called Enhancing Memory in Daily Life (E-MinD Life). As this was a newly developed intervention programme, identifying the key issues relating to its processes and feasibility before evaluating its clinical outcomes is recommended [50, 51]. Gathering the perspectives from both clinical experts and healthy older people was an essential first step in determining the feasibility of this proposed intervention prior to any attempt to administer the programme with a clinical client sample.

The study reviewed the feasibility, clarity, relevancy, and scheduling of the app-based cognitive strategy programme using a two-phase approach. The aim of phase 1 was to conduct an expert panel review of the E-MinD Life programme to (1) determine the feasibility, clarity, and relevancy of the programme and (2) review the formatting and scheduling of the intervention and eliminate irrelevant items from the programme and re-word items where necessary and add new items to the programme as recommended.

The aim of phase 2 was to pilot the E-MinD Life programme with a sample of older healthy people to (1) establish the acceptability of the programme and (2) assess the feasibility of the intervention and subsequent evaluation, the estimation of the likely recruitment and retention rates of participants, and the calculation of appropriate sample sizes for subsequent studies assessing the effectiveness of the programme. Later phases of the study will then be able to incorporate the findings of this study to modify and trial the app on a clinical sample of older adults with mild NCD and/or major NCD.

Approval to conduct the study was obtained from the Human Ethics Research Committee at Western Sydney University, Australia. All participants provided written informed consent prior to commencing the study. There were no specified ‘stoppage’ guidelines outlined to participants. Participants were able to withdraw from the study if they wished to do so.

### E-MinD Life programme

The E-MinD Life programme was designed to teach perceptual memory strategies to older people with mild NCD and early-stage major NCD so that they can use the app in their own homes to maintain or improve IADL performance.

Perceptual memory encoding techniques were applied to 12 common daily activities required for community living and built into the E-MinD Life programme on the app-based platform via Qualtrics (www.qualtrics.com). The 12 daily activities were chosen based on the results of a preceding study which investigated the most important, frequently performed and cognitively demanding IADL completed by older people that facilitate independent community living [52]. Activities included meal preparation, grocery shopping, using a telephone, making appointments, paying bills, and laundry activities.

The E-MinD Life programme was then developed to a point where trial, review, and feedback from the expert panel and the healthy older people were possible. The programme is outlined in a detailed diagram [see Additional file 1].

#### Phase 1: Expert panel review of the E-MinD Life programme

##### Participants

An expert panel of occupational therapists was convened to evaluate the content, feasibility, clarity, and relevancy of the E-MinD Life programme. Experts were skilled, registered occupational therapists who (i) were clinically experienced in acute or community-based practice in geriatrics and/or neurological practice, (ii) were familiar with occupational therapy interventions with older people, (iii) had an interest in cognitive strategies for older people, and (iv) had participated in research or quality assurance programmes in a related field. Experts meeting the inclusion criteria were invited to participate in the expert panel via email. Invitations were initially sent to networks known to the researchers, with permission given to email recipients to forward the invitation to other colleagues meeting the criteria, allowing recruitment to snowball [53]. Potential participants completed screening questions to confirm eligibility and gave consent prior to taking part in the study. A total of 12 expert therapists took part in this phase, which exceeds Lynn’s [54] suggestion that between five and ten experts are required to provide a reliable determination of the programme’s content. Participants were recruited over a 1-month period with a 2-week period between recruitment and commencement of the programme.

##### Procedure

Experts completed a demographic questionnaire which collected data on their age, gender, level of education, total years of occupational therapy practice, years of geriatric practice, years of neurological practice, and type of clinical setting in which they had worked or were working.

Prior to an expert review process, it is suggested that experts should have knowledge of the study’s hypothesis, key definitions, and the general goals of the programme being reviewed [55, 56]. Experts were given the option to either (a) attend a group session in which a live presentation was given by researchers (KL and NT) or (b) watch a pre-recorded presentation which was emailed to the participants. After watching the presentation, those attending in person were each provided with a printed instruction manual for the E-MinD Life programme and an iPad to trial the programme. Experts watching the video presentation were emailed a PDF version of the manual and app access to trial the programme on their own iPad. All experts were asked to review the instruction manual and programme prior to independently responding to a questionnaire provided as a printout at the group session or via email.

##### Expert panel review questionnaire

The questionnaire was adapted from a study conducted by Francois et al. [57] and adopted a Likert rating scale [53] to explore the elements of feasibility. The first author (NT) developed the questionnaire which was then reviewed and refined by the research team prior to use.

Experts rated the programme using a 5-point Likert rating scale that ranged from ‘poor’ (1) to ‘excellent’ (5), on 22 questions related to three key dimensions: feasibility, clarity, and relevancy. Feasibility was defined as the practicality and cost implications of the strategy (nine questions). Clarity was defined as the ease with which the experts felt that an older person with mild NCD might understand the instructions (seven questions). Relevancy was defined as how valuable, appropriate, and useful the expert therapists felt that strategy could be for improving IADL performance in older people (six questions). Experts were provided with the opportunity to make further comments on each question and suggest additional strategies and changes they felt were appropriate to consider during future programme development.

##### Statistical analysis

Expert characteristics were summarised by using mean and standard deviation for continuous variables and number and percent for categorical variables [Table 1]. For the expert panel review questionnaire, Likert scale responses were grouped into ‘broad agreement’ [comprising good (3), very good (4), and excellent (5)] and ‘broad disagreement’ [comprising fair (2) and poor (1)]. For each dimension (feasibility, clarity and relevancy), the number of responses in each grouping (broad agreement and broad disagreement) was summed up and then divided by the total number of responses, thus obtaining a percentage ‘broad agreement’ and ‘broad disagreement’ for each dimension.

Feasibility, clarity, and relevancy (i.e. each dimension) were considered acceptable if there was > 79% agreement, questionable if there was 70–79% agreement, and unacceptable if there was 69% agreement [58].

Open-ended responses pertaining to each dimension were collated and categorised qualitatively. The constant comparative approach of open coding of qualitative data was used to group similar responses into categories [59, 60] relating to four aspects: feasibility, clarity, relevancy, and suggested changes to the E-MinD Life programme. Responses to each open-ended question for each participant were coded by the first author (NT) and confirmed by the second author (RB). Codes were then compared between experts to identify similarities and differences between responses to develop provisional categories [59, 60]. The initial categorical groupings and titles were further discussed with the research team (NT, RB, KL) and renamed and amended until a consensus was reached.

#### Phase 2: Field test—feasibility and acceptability of the E-MinD Life programme with healthy older people

Phase 2 ran concurrently with phase 1 of this study.

##### Participants

Community-dwelling older people aged 65 years and over were recruited via convenience sampling techniques from two independent living retirement villages. The time and associated costs of implementing the E-MinD Life programme with each individual participant as well as the ethical considerations that participants may be exposed to an ineffective intervention have to be considered as part of the determination of the adequate sample size for preliminary feasibility studies [61]. Furthermore, previous studies indicate that six participants are adequate to assess the feasibility of intervention programmes [62]. The sample size is selected due to feasibility rationale, as per Julious [63] who discussed the precision of the mean and variance and regulatory considerations leading to a suggested sample size of 12 [63]. A sample of nine to 12 participants has therefore been chosen in this study.

A face-to-face information and recruitment group presentation was held by one of the researchers (NT) at each of the independent living retirement villages. Participant information sheets with details about registering for the project were provided to all attendees. Attendees self-reported to be cognitively healthy with no previous psychiatric or memory disorder or other neurological illnesses that may affect their cognition. Potential participants contacted the researcher (NT) to express interest and were subsequently visited in person by the researcher (NT) and screened for inclusion by administering the Mini-Mental State Examination, 2nd edition, standard version (MMSE) [64] and a 15-item Geriatric Depression Scale—short form (GDS) [65]. Participants required a MMSE greater than 24 and a GDS score less than five. Participants were not required to have previous experience of using an iPad nor were they required to have internet access. There were no changes made to the eligibility criteria after the commencement of the study. Participants were recruited over a 2-month period. There was also an additional 2-week period between providing consent and commencement of the programme. Participants were from a two-arm randomised control trial. This current study reports the group of participants who received the current perceptual-based E-MinD Life programme.

##### Procedure

Each participant was provided with an iPad with the app-based E-MinD Life programme installed. The intervention consisted of three 60-min sessions per week (one individual face-to-face therapist led session and two self-administered sessions) over 9 weeks. Therapist-led sessions were conducted in each participant’s home, facilitated by a research assistant with a background in occupational therapy. The time to complete the 9-week programme was extended to 12 weeks to allow for interruptions and changes in participants’ schedules. The intervention was stopped once participants completed the 9-week intervention or after a 12-week period.

##### Measures

***Baseline characteristics*** Participant baseline demographic data was obtained via an interview with the primary researcher. The Lawton Instrumental Activity of Daily Living Scale [66] was administered to provide baseline information on independent living skills.

***Feasibility measures***

*Recruitment and retention* The recruitment rate was quantified as the percentage of all participants who satisfied the inclusion criteria and agreed to participate. This was calculated on the conversion of potential participants who were invited to participate to then being enrolled in the programme. This study considered recruitment success to be 10% of potential participants agreeing to be enrolled in the study. The retention rate was quantified as the percentage of participants who commenced the E-MinD Life programme and remained in the study at the end of the programme.

*Attendance and completion of therapist-led and self-administered sessions* Attendance at the 9-week therapist-led sessions was recorded and a percentage attendance rate was calculated. By reviewing the participants’ activities in the E-MinD Life programme app, each self-administered session was judged as ‘completed’, ‘partially completed’, or ‘not attempted/incorrect session completed’ and scored 1, 0.5, or 0 points, respectively. The scores were summed and divided by 18, the total number of self-administered sessions possible, to produce a self-administered completion rate for each participant. ‘Incorrect session completed’ occurred when the participant had difficulty in selecting the appropriate session, for example, completing session two twice instead of completing the third session for that week. In addition, ‘extra completed’ sessions occurred when a participant completed a session more than the required one time. These ‘extra completed’ sessions did not contribute to the self-administered completion rate.

Reasons for not completing sessions (either mode) were recorded by the research assistants implementing the programme.

*Time taken to complete the self-administered sessions* The duration of the 18 self-administered sessions was recorded, and the average duration of the completed sessions was calculated for all participants. If the session was partially completed, the duration was omitted from the calculation.

***Acceptability measures*** Following the completion of the 9-week programme, participants completed an acceptability questionnaire with the assistance of a research assistant. The 39-item acceptability questionnaire covered four areas: (1) overall programme, (2) therapist-led sessions, (3) self-administered sessions, and (4) the computer programme and iPad use. In each area, questions across four dimensions were adopted from Francois et al. [57]. These dimensions were (1) perceived effectiveness—how participants perceive the programme allowed them to acquire, practise, and master the cognitive strategies (13 items); (2) relevancy—views regarding the appropriateness of the programme for older people and applicability of the daily activities (9 items); (3) convenience—participant perceptions of how easy it was to participate in the programme (7 items); and (4) clarity—participant perceptions of whether the programme was logical and easy to understand, including if instructions were appropriate to complete the required activities (10 items). Participants rated each question on a 4-point Likert scale [completely disagree (1), disagree (2), agree (3), completely agree (4)]. Participants were encouraged to provide additional suggestions in relation to each dimension and asked to rate (1) if they would recommend the programme to other older people 65 years and older and (2) if they would use the programme if it was part of an occupational therapy intervention and be satisfied with the therapy provided.

There were no changes made to the methods of assessment/measurement tools after the commencement of the study.

##### Statistical analysis

The demographic characteristics of participants were summarised by using the mean and standard deviation for continuous variables and number and per cent for categorical variables. Data from each questionnaire were entered into a spreadsheet and tabulated. Likert scale responses were grouped into ‘broad agreement’ [agree (3) and completely agree (4)] and ‘broad disagreement’ [disagree (2) and completely disagree (1)]. For each dimension (perceived effectiveness, relevancy, convenience, and clarity), all applicable questions that were given a rating of ‘3’ or ‘4’ (i.e. broad agreement) by participants were summed and divided by the total number of responses in the dimension. Participant recruitment rates, adherence to the programme, duration of sessions, and barriers to the implementation of the programme were summarised and reported as frequencies and proportions or as free text. Statistical analysis occurred once all participants had completed the intervention. No interim analyses were conducted.

The E-MinD Life programme will be considered feasible for inclusion into a future trial design study to determine the programme’s effectiveness if the following progression criteria are reached:

1. More than 10% of potential participants approached during the recruitment phase of the study provide consent to participate in the study.
2. More than 80% of participants who commence the programme complete the 9-week programme.
3. Participants attend at least 80% of the therapist-led sessions and self-administered sessions.
4. The average time taken for each session is no more than an hour to ensure the programme can be incorporated into clinical practice.
5. There is more than a 79% broad agreement rate for (1) feasibility, (2) clarity, and (3) relevance among the expert panel of occupational therapists who reviewed the programme.
6. There is more than a 79% broad agreement rate among the older adults who trialled the E-MinD Life programme for each of the following: (1) perceived effectiveness, (2) relevance, (3) convenience, and (4) overall programme clarity.
7. At least 79% of older adults who trialled the E-MinD Life programme would either recommend the programme to others or would use the programme again in the future if shown to be effective.

For any results falling below the above progression criteria, the research team will discuss possible changes in recruitment, programme delivery and use of outcome measures prior to any future study examining the programme.

## Results

### Phase 1: Expert panel review of the E-MinD Life programme

A total of 12 occupational therapists were recruited in the expert panel review. The experts ranged in age from 26 to 56 years, with all recruited experts being female. A quarter (*n* = 3) of the experts had a PhD level qualification, another quarter (*n* = 3) had a masters level qualification, and the remaining experts (*n* = 6) had a bachelor level qualification. Experts had between 5 and 33 years of experience as an occupational therapist. Characteristics of the expert panel are outlined in Table 1.

**Table 1:** Experts’ characteristics (expert panel)

*N* = 12	
Female, *n* (%)	12 (100%)
Age (years, mean ± SD)	36.58 ± 8.20
Level of qualification, *n* (%)	
Bachelors	6 (50%)
Masters	3 (25%)
PhD	3 (25%)
Years of occupational therapy practice (years, mean ± SD)	13.67 ± 8.96
Years of geriatric practice (years, mean ± SD)	7.02 ± 7.28
Years of neurological practice (years, mean ± SD)	1.12 ± 2.33
Current caseload/setting, *n* (%)	
Acute care	2 (16.67%)
Rehabilitation and psychogeriatric	1 (8.33%)
Community	3 (25%)
Acute care and community	3 (25%)
Tertiary education	3 (25%)

Experts indicated broad agreement across all three dimensions of feasibility, clarity, and relevancy. The combined broad agreement score for the feasibility was 85% (mode = 4 completely agree). The combined broad agreement score for relevancy was 90% (mode = 4 completely agree). The combined broad agreement score for clarity was 85% (mode = 4 completely agree).

The extended responses from the expert panel showed that the E-MinD Life programme had both favourable (strengths) and unfavourable (opportunities) components, noting potential updates for future programme development. These components were further categorised according to feasibility, clarity and relevancy.

#### The current strengths of the E-MinD Life programme

Seven categories were constructed from the qualitative data in relation to the strengths of the programme, and these are outlined in Additional file 2 with direct quotes from experts. The first strength category, *the occupational therapist cannot be removed from the therapy process*, revealed that experts felt that, although the programme was designed to be self-administered, the occupational therapist continues to play an important role in the therapy process and implementation of the programme. The second strength category, *E-MinD Life is within the scope of occupational therapy practice*, shows that the experts believed that cognitive training and teaching of cognitive strategies are within the scope of practice for occupational therapists, and IADL performance is quintessential occupational therapy. The third strength category, *E-MinD Life includes relevant IADLs for community living*, highlights experts’ agreement that the IADLs are relevant for community living and replicate everyday life. The fourth strength category, *if found effective, occupational therapists would use E-MinD Life for therapy*, indicates experts welcome the opportunity to enhance current therapy with an app-based programme and would support the use of the E-MinD Life programme following further research into the effectiveness of the programme. The fifth strength category, *duration and frequency of sessions are appropriate*, revealed the experts found a 45–60-min programme administered three times a week to be sufficient for achieving the desired outcome of the programme and one therapist-led session per week would be feasible in clinical practice. The sixth strength category, *repetition and practice form a strong foundation for E-MinD Life*, highlights the experts’ opinions that repeated practice of a task is essential in cognitive interventions and this was considered in the development of the E-MinD Life programme. The final category, *the videos are clear and support the programme*, refers to the experts highlighting the videos of the 12 entire IADLs and the videos of each step of these IADLs played a role in making the programme engaging and assisted in presenting the IADLs in a visual format to support the visualisation strategy taught during the programme.

#### Opportunities for future development of the E-MinD Life programme

Nine categories were generated from the qualitative data in relation to opportunities for the future development of the E-MinD Life programme. These categories are outlined in Additional file 3 with illustrative quotes. The first category, *flexibility of therapist-led session should be considered*, highlights the experts’ views regarding future consideration of an individualised approach as some older people may require more than one therapist-led session per week to learn the programme and cognitive strategy. Experts felt this was something the therapist should be able to adapt following the initial therapist-led session based on how the client performed during the session. The second category, *adaptations of the programme for different clinical settings could support the feasibility of the programme,* highlights the experts’ concern regarding the feasibility of the programme across various occupational therapy settings such as community-based, residential aged care, and rehabilitation services. Experts felt the duration of the programme and the funding of staff required for the administration of the programme could be a barrier to implementing the E-MinD Life programme. The third category, *traditional methods of repetitive task-orientated practice may compliment the app-based therapy*, refers to the experts acknowledging the foundation of ‘doing’ that underpins occupational therapy practice. The fourth category, *the ‘drag and drop’ function is not intuitive for older people*, relates to the experts’ concerns that many of the activities in the E-MinD Life programme require knowledge of ‘drag and drop’. Experts suggest that more training and prompting on this function is required to support older people being able to independently administer the programme. The fifth category, *improvements to the visual design and visual prompts to enhance clarity and improve the user experience*, is related to the formatting of the E-MinD Life programme, with several experts reporting improvements in font size, contrasting of colours and increased image sizes are required to take into consideration visual needs of older people. The sixth category, *feedback and progress updates would be beneficial for older people and therapists*, expresses experts’ encouragement to add real-time feedback to provide reassurance to older people using the programme. Experts suggested a progress bar to highlight how far through the session a person is as this may improve the completion rates of each session. The seventh category, *a tailored and individualised approach with an expansion of IADL will further support the use of the programme in occupational therapy services*, acknowledges the expert’s consideration into the diversity of daily activities completed by older people and the variation in how these are completed among individuals. Experts encourage further development of the E-MinD Life programme to include additional IADLs and the ability for therapists to adapt the steps of the IADLs to match the preferences of their clients. The eighth category, *concerns for older people with limited previous exposure to technology*, reflects on the fundamental concept of person-centred practice used in occupational therapy. Experts felt that those with limited previous exposure to app-based programmes would require additional support throughout the 12-week programme. The final category, *the current version of E-MinD Life is not for everyone*, acknowledges that while therapists may have a willingness to discover what technology has to offer, the current version of the E-MinD Life is not suitable for all older people. As an example, clients from culturally and linguistically diverse backgrounds or those experiencing sensory deficits may be unlikely to be able to use the programme in its current design.

### Phase 2: Field test—feasibility and acceptability of the E-MinD Life programme

#### Participants

The sample included nine participants. Participants ranged in age from 68 to 86 years and five identified as male. GDS scores ranged between 0 and 4. Two participants scored 0, one participant scored 4, and the remaining participants scored 1. All participants’ MMSE scores were within the ‘normal cognition’ range. The Lawton IADL scores indicated participants were independent in most activities. Participant demographics and baseline characteristics are described in Table 2.

**Table 2:** Participant characteristics

	*n* = 9
Female, *n* (%)	4 (44.44%)
Age (years, mean ± SD)	78.11 ± 4.81
Level of education	
Secondary education, *n* (%)	4 (44.44%)
Higher education (tertiary)	1 (11.12%)
Higher education (technical college)	4 (44.44%)
Living arrangements	
Living alone	2 (22.22%)
Living with spouse	7 (77.78%)
MMSE (mean ± SD)	28.78 ± 1.09
Lawton IADL (mean ± SD)	7.78 ± 0.67

*SD* Standard deviation, *MMSE* Mini-Mental State Examination, *Lawton IADL* Lawton Instrumental Activity of Daily Living Scale

#### Feasibility

##### Recruitment and retention

Of the 54 older people who attended the information sessions across the two independent living retirement villages in greater Western Sydney, Australia, 23 agreed to participate in the study. Three of these people did not meet the selection criteria based on the MMSE score. Excluding the three participants that did not meet the inclusion criteria, the recruitment rate was 37.04%. Nine participants were allocated to this study and all agreed to participate. There was a 100% retention rate over the duration of the study.

##### Attendance and completion

Participants attended between 6 (66.67%) and 9 (100%)’ of the therapist-administered sessions. Six participants (66.67%) completed all 9 therapist-led sessions, with three doing so in 9 weeks, two requiring 10 weeks, and two requiring 12 weeks. The average number of therapist-led sessions attended was 8.33 (92.56%) (SD = 1.12) out of the possible 9 sessions.

Participants attempted between five (27.78%) and 24 (133.33%) of the 18 self-administered sessions. The average number of sessions attempted was 13 (SD = 6.73) out of the 18 scheduled sessions. Five participants (55.56%) completed all 18 sessions. Two participants attempted more than the scheduled 18 sessions.

The reasons provided for cancelling or rescheduling the therapist-led sessions included being unwell or attending medical appointments (*n* = 3), travel and holidays (*n* = 4), caring for spouse or grandchildren (*n* = 4), or other commitments such as social and family engagements (*n* = 3). Reasons provided for non-completion of the self-administered sessions were similar to those provided for the therapist-led sessions with the addition of difficulties navigating and using the iPad and technical difficulties with the programme.

##### Duration

The mean duration of the therapist-administered and self-administered sessions was 67.92 min (SD = 10.33, range 50–85) and 34.25 min (SD = 16.36, range 9–70), respectively.

The average duration of the self-administered sessions reduced over the course of the programme from 48 min for block one, 41 min for block two, and 32 min for block three.

#### Acceptability

**Fig. 1:**
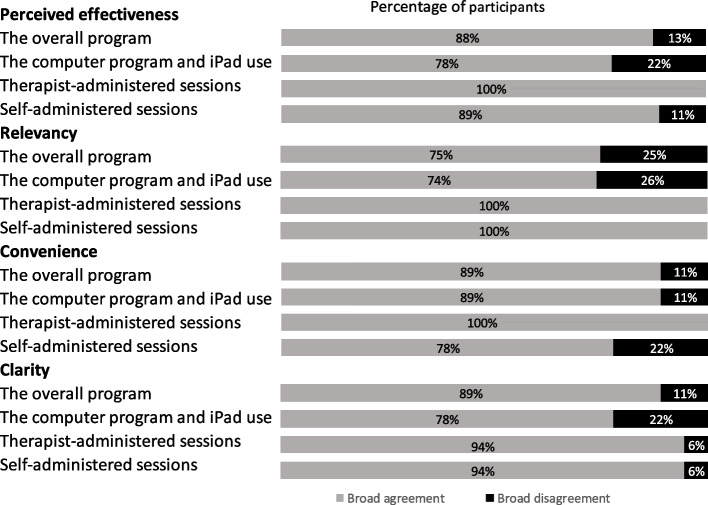
Older people’s acceptability of the E-MinD Life programme

Participants perceived the overall programme to be effective in allowing them to acquire, practise and master the cognitive strategies, evidenced by a broad agreement of 88% in responses to questions about acceptability. Three-quarters (75%) of the responses indicated participants rated the overall programme to be appropriate for older people and thought the daily activities were applicable. Eighty-nine per cent of responses noted that the overall programme was easy to participate in, logical and easy to understand. The results for the four dimensions (perceived effectiveness, relevancy, convivence, and clarity) across the four areas (overall programme, therapist-led sessions, self-administered sessions, and the computer programme and iPad use) are presented in Fig. 1.

Eight of the nine participants (88.9%) would recommend E-MinD Life to others and rated broad agreement regarding their intention to use the programme in the future if proven to be an effective cognitive strategy for the maintenance of cognition and IADL performance.

There were no unintended consequences, harms, or effects identified by the research team or reported by participants during the study.

## Discussion

In this study, the feasibility and acceptability of a cognitive strategy programme called E-MinD Life were assessed. The results showed that the E-MinD Life programme, using perceptual memory encoding, was feasible and acceptable among an expert panel and healthy older people.

Phase 1 of this study used an expert panel to determine the feasibility of the programme to complement traditional methods of occupational therapy with older people. The feedback from the expert panel advisory group during phase 1 of the study raised three points for discussion.

First, the combined broad agreement score for the feasibility of the programme was 85.0%, demonstrating the experts perceived the programme to be a practical and cost-effective strategy for therapy. A key strength of the E-MinD Life programme also supporting the feasibility of the programme and constructed from the qualitative analysis was *duration and frequency of the sessions are appropriate*. Another key strength constructed from the qualitative analysis and supporting the feasibility of the E-MinD Life programme was *if found effective, occupational therapists would use E-MinD Life for therapy*. These findings indicated that therapists will be able to use a programme such as E-MinD Life within the current time constraints of therapy services and are willing to try new intervention approaches. This is an important finding for the E-MinD Life programme promoting evidence-based healthcare and ensuring available interventions are practical and practicable within a given context, consistent with the JBI model of evidence-based healthcare [67].

Second, the combined broad agreement score for the relevancy of the programme was 90.0%, indicating the experts determined the programme to be a valuable, appropriate, and useful strategy with the potential for improving IADL performance in older people. Key strengths of the E-MinD Life programme further supporting the relevancy of the programme and constructed from the qualitative analysis were *E-MinD Life includes relevant IADLs for community living* and *E-MinD Life is within the scope of occupational therapy practice*. This aligns with the JBI model of evidence-based healthcare in which interventions should be appropriate and relate to the context in which care is provided [67].

Third, the combined broad agreement score for the clarity of the programme was 85.0%, indicating the experts felt that an older user with mild NCD would be able to understand the instructions and complete the programme. However, findings from the qualitative analysis suggest several formatting changes in future iterations of the programme are required to enhance the useability of the programme. This is an important finding as it is now possible to include additional prompts and design features within the E-MinD Life programme to assist older people with mild NCD to successfully engage in the programme while avoiding frustration due to programme design. Aligning with the expert’s perspectives regarding feasibility, and to further improve adherence to the programme, future iterations of E-MinD Life will consider a more flexible approach to the type of session. The participant and the therapist should be able to adjust the ratio of therapist-led to self-administered sessions to suit the participant’s individual needs.

Overall, the results of the expert panel review indicated occupational therapists find the programme to be feasible and could potentially enhance traditional methods used in occupational therapy practice.

Phase 2 of this study aimed to further examine the feasibility of the E-MinD Life programme by examining older people’s recruitment, retention, attendance, and completion to the programme to prepare for subsequent studies assessing the effectiveness of the programme. Phase 2 also aimed to explore how older people perceived the effectiveness, relevancy, clarity, and convenience of the programme. The findings from phase 2 raised several points for discussion.

First, although recruitment rates were low, there were no dropouts over the duration of the study. A meta-analysis of computerised cognitive training on cognitive outcomes in mild cognitive impairment reported dropout rates ranging from 0 to 32% [68]. Similarly, a meta-analysis of the everyday impact of cognitive interventions in mild cognitive impairment reported retention rates ranging from 64 to 100% [69]. Based on this information, and considering we recruited healthy older people, we determined a successful retention to be defined as less than 20% attrition rate for those participants who commenced and successfully completed the 9-week programme. For future studies, we will consider staggered recruitment strategies to overcome the growing demand for older people to participate in research and the difficulties associated with recruitment in a city saturated with academic and research institutes. This staggered recruitment strategy has been shown to be successful in other studies [70].

Second, attendance to the programme suggests the schedule of the E-MinD Life programme was feasible for older people. Our initial plan outlined that participants should complete the 9-week programme over a period between 9 and 12 weeks. The buffer period of 3 weeks allowed for participants to complete the programme even if they experienced illness, had medical appointments to attend, or went on vacation. However, Joosten et al. [71] considered an appropriate adherence rate for older people with mild NCD enrolled in a cognitive behavioural group to be attendance at 80% of sessions. Although the average number of therapist-led sessions attended was 91%, completion of the self-administered sessions was lower at 74%. Low adherence rates can reduce the generalisability and validity of controlled trials or decrease the power of the study and lead to an underestimation of the efficacy of an intervention. In future studies, an attempt to minimise the number of participants who may experience challenges with adherence will need to be made. This can be achieved by (a) attempting to minimise the number of participants who may have difficulties adhering to the programme due to travel plans, caring responsibilities, volunteer commitments, or medical conditions/appointments by identifying them prior to randomisation or during an initial ‘trial’/ ‘run-in’ period of the intervention [72, 73]. Participants should be given the opportunity to trial the intervention prior to committing to the 9-week duration; and (b) ensuring participants are well informed and expectations regarding the time commitment of the programme are explicitly discussed prior to commencement [72].

Furthermore, an interesting finding was that the self-administered sessions were shorter than the therapist-led sessions. This could have been due to the ‘teaching/learning approach’ of the therapist-led sessions requiring more time. However, it may also be due to participants completing the tasks of the programme based on previous attempts without necessarily applying the taught cognitive strategy. Future iterations of the programme will need to consider how to encourage participants to continue to apply the taught cognitive strategy and only allow participants to progress from each task after an appropriate amount of time has been given to implement the visual imagery techniques. Additionally, to further improve adherence to the programme, when examining the effectiveness of the programme, it would be beneficial to determine if shorter sessions achieve similar results. If shorter sessions are possible, participants may be more likely to adhere to the programme.

Regarding the relevancy of the programme, only 75% of participants thought the daily activities were applicable to older people. This result may be due to ‘healthy’ older people having intact IADL performance and being unaware of what challenges they may experience if they were to be diagnosed with mild NCD. Alternatively, the result may also be due to over half the participants being male and traditional gender roles may still be present among older people. This is a consideration for further development of the E-MinD Life programme as older people may disengage from interventions if they are not meaningful and relevant to them. Findings from Fox et al. [74] indicated older people were more likely to give up or perform instrumental activities less, with 51% of their sample stating this was due to ‘no interest’ in the activity. Exploring an individualised approach where older people can self-select from a range of IADLs will be considered in the future development of the programme.

Overall, the results from phase 2 demonstrated that a self-administered approach in addition to therapist-led sessions was feasible and was characterised by high participant acceptability.

Developing and evaluating interventions targeted at cognition and IADL performance is a high priority for older people with and without mild NCD. This study followed the first two stages outlined by the Medical Research Council’s framework for developing and evaluating complex interventions [75]. These two stages involved (1) the expert panel review of an intervention and (2) assessment of the feasibility of the intervention by running the intervention on healthy older adults. This study was strengthened by the researchers sharing the literature and background on the topic with the experts who provided feedback to inform the development of the programme. Furthermore, this study involved the consumer’s perspective, which may enhance the future development of the programme. The involvement of these stakeholders may assist with overcoming practical obstacles to the evaluation and implementation of the programme in future trials.

### Implications for future research

This study demonstrated the feasibility and acceptability of the E-MinD Life programme to healthy older adults. The effectiveness of the E-MinD Life programme for improving cognition or IADL performance has not yet been established. In accordance with the Medical Research Council’s framework for developing and evaluating complex interventions [75], future research is required to determine if the adopted E-MinD Life programme using perceptual memory strategies has functional and cognitive benefits for healthy older people and if the programme is able to delay functional deterioration during the cognitive decline. Further research is required. Furthermore, the effectiveness of the E-MinD Life programme with older people with mild NCD, the targeted population for the programme is not known. Further research examining the feasibility and acceptability of perceptual memory strategies for IADL performance among older people with mild NCD is needed.

### Study limitations

Several key limitations need to be considered in relation to this study. Firstly, the sample size was small thus limiting the generalisation of results to a broader context and preventing the use of statistical analysis to examine individual factors within the sample. Secondly, while participants with mild NCD were a targeted population for the programme, all participants in this study were healthy older adults. Therefore, once the programme is amended based on the results of this study, further studies are required to determine the acceptability and feasibility in older adults with mild NCD. Thirdly, the effectiveness of the programme for improving cognition or IADL performance has not yet been examined, as this study only rated perceived effectiveness. Additionally, E-MinD Life places emphasis on enhancing memory skills necessary for IADL performance and developing strategies for memory retention. As a result, future trials involving older adults with mild NCD, E-MinD Life is less likely to benefit individuals with impairments in cognitive domains other than memory and learning. Finally, this study excluded older people with any self-reported previous psychiatric disorders. As the prevalence of depression and anxiety are high among older people [76], the generalisability of the results may be limited.

## Conclusion

Occupational therapy offers a unique approach to helping older people remain living in the community. These findings support the feasibility of an accessible intervention mode for older people that could compliment traditional occupational therapy services. The E-MinD Life programme was acceptable and promising in terms of its perceived effectiveness on functional cognitive problems. Recruitment, retention, and completion rates were acceptable indicating the programme shows promise for inclusion into randomised control trials to determine the effectiveness of the programme. Taking into consideration the findings from this study, it is recommended E-MinD life is updated prior to the effectiveness of the programme being determined. It is recommended the updated E-MinD life is trialled not only with healthy older people but also with older people with mild NCD.

## Supplementary Information

**Additional file 1. **Enhancing Memory in Daily Life (E-MinD Life): Basic study design and programme outline. A figure and a table with figures outlining the scheduled sessions involved in the study and the steps completed by participants during E-MinD Life [77].

**Additional file 2: Table S2. **Summary of expert’s perspectives on E-MinD Life: Current strengths of E-MinD Life. Categories constructed from the qualitative data in relation to strengths of the programme with direct quotes from the experts.

**Additional file 3: Table S3. **Summary of expert’s perspectives on the E-MinD Life Programme: Opportunities for future development of E-MinD Life. Categories generated from the qualitative data in relation to opportunities for the future development of the E-MinD Life programme with illustrative quotes.

## Data Availability

The datasets used and/or analysed during the current study are not yet publicly available but are available from the corresponding author upon reasonable request.

## Abbreviations

E-MinD Life: Enhancing Memory in Daily Life
BADL: Basic activities of daily living
GDS: Geriatric depression scale
IADL: Instrumental activities of daily living
NCD: Neurocognitive disorder
MMSE: Mini-Mental State Examination 2 (standard version)
