# Supplementary material for: The feasibility and acceptability of an app-based cognitive strategy training programme for older people
Source: Pilot Feasibility Stud. 2023 Jun 30;9:109. doi: 10.1186/s40814-023-01334-x (PMC10311870; doi:10.1186/s40814-023-01334-x)
Supplement: Supplementary file 1 — Additional file 1. Enhancing Memory in Daily Life (E-MinD Life): Basic study design and programme outline. A figure and a table with figures outlining the scheduled sessions involved in the study and the steps completed by participants during E-MinD Life [77]. [file 40814_2023_1334_MOESM1_ESM.docx]

**Additional File 1 – Enhancing Memory in Daily Life (E-MinD Life): Basic study design and programme outline**

In the app, the 12 daily activities were divided into three blocks, each containing four activities. Blocks were broadly classified under ‘meal preparation and clean-up activities’, ‘laundry activities’, and ‘communication and community activities’. Each block was then designed to be the focus of a three-week perceptual memory encoding strategy learning period, with the total programme length being nine weeks.

The perceptual memory encoding strategies used in E-MinD Life involved the use of visual imagery and the method of loci. As noted previously, visual imagery involves the creation and encoding of images by a method where activities, behaviours or performance are practiced in the mind as though they were being performed. While the method of loci places these images into context of the environment to prompt memory [18]. To achieve this in the app, each of the 12 daily activities were filmed and a short video of each created. Naturalistic settings (such as kitchens and laundries) were used for the performance of each daily activity, and an occupational therapy student was filmed performing each activity. In addition, each activity was broken down into six photographed steps based on activity analysis and task breakdown methods commonly used by occupational therapists [77].

**Equipment**

Each participant was provided with an iPad with the cognitive strategy programme installed.

**Frequency and duration**

The intervention was planned to consist of three 60-minute sessions per week over a 9-week duration (Figure 1). Participants were given up to 12 weeks to finish the 9-week programme to allow for interruptions and changes in participants' schedules.

**Figure 1.** Basic study design

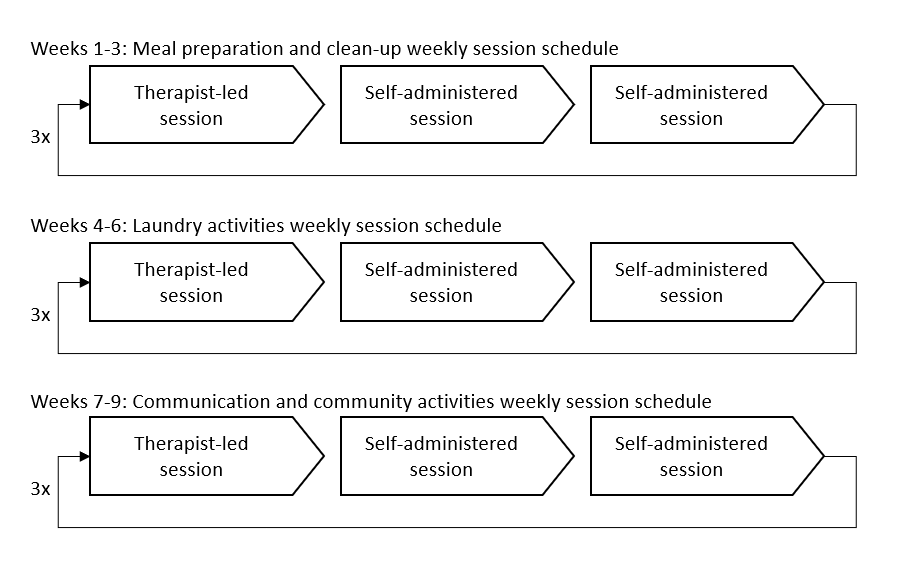


**Therapist-led and self-administered sessions**

Each week entailed one individual face-to-face therapist-led session and two self-administered sessions per week. Therapist-led sessions were administered by a research assistant with a background in occupational therapy at the participant’s home. These sessions were tailored to each participant’s learning needs while employing a standardised approach for all participants across the sessions.

Each therapist-led session commenced with a recap of the previous session and discussion about the self-administered sessions, to address any issues that had arisen. Following the weekly therapist-led session, participants completed the two self-administered sessions at a time that was convenient to their weekly schedule. During the self-administered session, the participant would complete the same activities as those practiced during the therapist-led session utilising the memory techniques taught during the therapist-led sessions (Figure 2).

**Figure 2.** Outline of the steps completed during the E-MinD Life Programme

| **Step 1** - Programme introduction covered four daily activities in the session. | 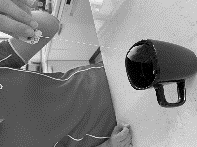 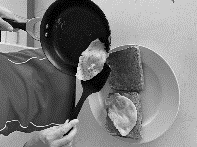 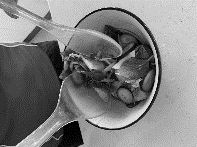 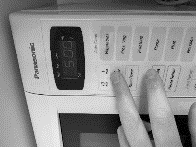  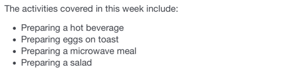 |
| --- | --- |
| **Step 2** - Participants reporting the number of completed individual activities since the prior session. | 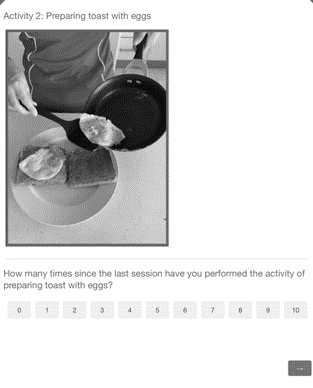  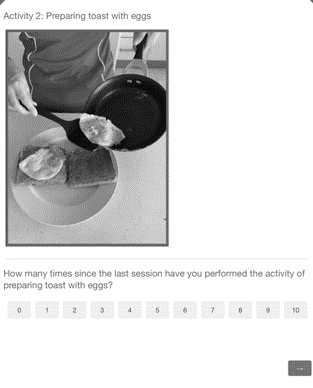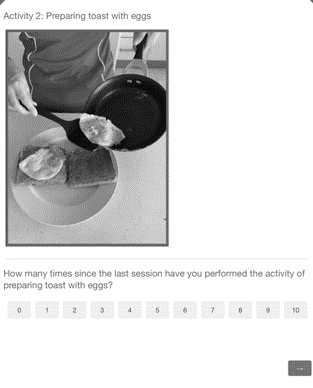 |
| **Step 3** - Participants watching a video of a daily activity being performed. | 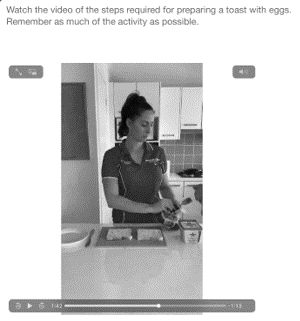  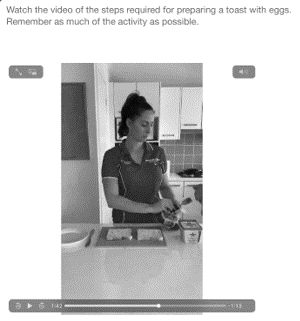 |
| **Step 4** - Participants verbalising the photographically presented steps of a daily activity and applying the memory strategies taught. | 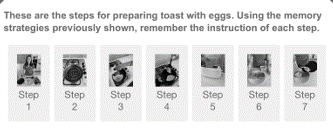 |
| **Step 5** - Participants imagining performing a daily activity in locus solely with the support of an auditory aide for each step of the daily activity. | 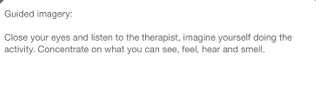 |
| **Step 6** - Participants identifying the appropriate environment for the performance of a given daily activity.  **Step 7** - Participants visualising themselves performing the relevant daily activity in the identified environment. | 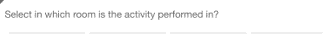  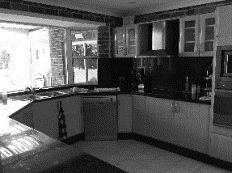 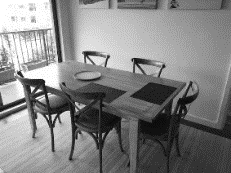  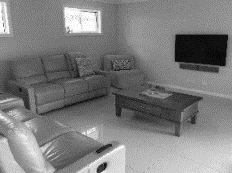 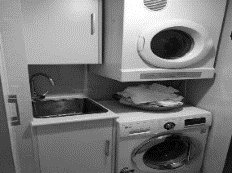 |
| **Step 8** -  Participants presented with a sequence of photographed steps, some of which were removed.  The programme prompted the participant to select the appropriate step from several photographs displayed on the screen. Each time a photograph was selected and replaced back into the sequence the participant was prompted to visualise themselves completing the step.  Over time, difficulty was ramped up by increasing the number of steps removed from the daily activity | 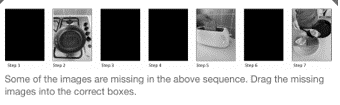  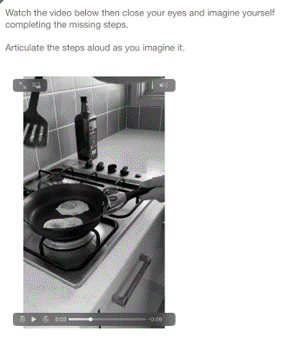  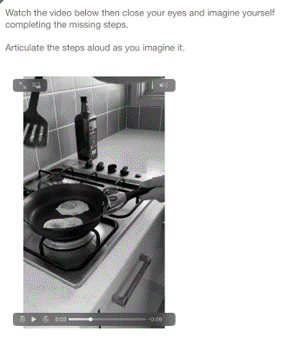 |
| **Step 9** - Participants rewatching the entire video of the daily activity once again, being encouraged to visualise and themselves performing the daily activity in locus. | 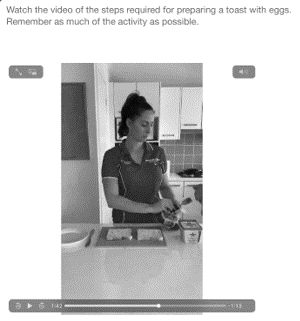  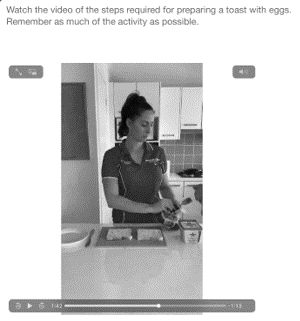 |
| Participants completing steps 2 to 9 for the 4 daily activities described in Step 1. | |
