# Supplementary material for: The feasibility and acceptability of an app-based cognitive strategy training programme for older people
Source: Pilot Feasibility Stud. 2023 Jun 30;9:109. doi: 10.1186/s40814-023-01334-x (PMC10311870; doi:10.1186/s40814-023-01334-x)
Supplement: Supplementary file 2 — Additional file 2: Table S2. Summary of expert’s perspectives on E-MinD Life: Current strengths of E-MinD Life. Categories constructed from the qualitative data in relation to strengths of the programme with direct quotes from the experts. [file 40814_2023_1334_MOESM2_ESM.docx]

| **Additional Item 2:** Summary of expert’s perspectives on E-MinD Life: Current strengths of E-MinD Life | |
| --- | --- |
| **Category** | **Illustrative Quotes** |
| **Feasibility** | |
| If found effective, occupational therapists would use E-MinD Life for therapy. | Expert 8: I would definitely recommend it [E-MinD Life] if the research shows positive outcomes and good feedback from participants around feasibility… I think it’s a great way of providing additional therapy to clients without the need for a face-to-face therapist.  Expert 11: Definitely, [recommend E-MinD Life] there is a paucity of evidence-based interventions in this space. |
| Duration and frequency of sessions are appropriate. | Expert 5: 60 minutes is sufficient to teach as well as provide ongoing prompting and encouragement for people with dementia.  Expert 9: Brilliant time allocation and continuity from my experience. |
| **Clarity** |  |
| Repetition and practice form a strong foundation for E-MinD Life. | Expert 5: Good enough repetition by themselves [older person] and then review with therapist is a great idea.  Expert 11: Current evidence supports multiple sessions over multiple weeks, so this is excellent. |
| The videos are clear and support the programme. | Expert 11: Easy to use, good videos of task performance broken down into appropriate, logical and relevant steps. Based on theoretical constructs of encoding.  Expert 10: The video is well put together and clear. |
| **Relevancy** |  |
| E-MinD Life is within the scope of occupational therapy practice. | Expert 9: Very occupation based. These tasks tend to be very meaningful and valuable to clients as well.  Expert 11: Occupational therapists are well equipped and experienced in provided home based assessment and interventions. The naturalistic environment is best for this type of intervention; however, a clinical setting is appropriate as well. |
| E-MinD Life includes relevant IADLs for community living. | Expert 1: A good variety. Very relevant tasks. Hot beverage, eggs on toast, and microwave meal.  Expert 12: Highly relevant, includes ones [IADLs] that are usually safety concerns and depict whether the person needs services or alternative living arrangements. |
| The occupational therapist cannot be removed from the therapy process. | Expert 5: Initial AX. [assessment] to be completed prior to intervention. It would have a better insight if OT [occupational therapist] responds with visual or verbal/word cues.  Expert 9: Education from the therapist would be essential. |
