# Supplementary material for: The feasibility and acceptability of an app-based cognitive strategy training programme for older people
Source: Pilot Feasibility Stud. 2023 Jun 30;9:109. doi: 10.1186/s40814-023-01334-x (PMC10311870; doi:10.1186/s40814-023-01334-x)
Supplement: Supplementary file 3 — Additional file 3: Table S3. Summary of expert’s perspectives on the E-MinD Life Programme: Opportunities for future development of E-MinD Life. Categories generated from the qualitative data in relation to opportunities for the future development of the E-MinD Life programme with illustrative quotes. [file 40814_2023_1334_MOESM3_ESM.docx]

| **Additional Item 3** Summary of expert’s perspectives on E-MinD Life: Opportunities for future development of E-MinD Life | |
| --- | --- |
| **Category** | **Illustrative Quotes** |
| **Feasibility** | |
| Flexibility of therapist-led session should be considered. | Expert 6: Could benefit from two therapist led sessions: 1) teaching and 2) pt. [patient] doing with supervision. Number of therapist-led sessions should be flexible based on pt.'s [patients’] needs.  Expert 11: It may depend on how the participant went in the first session with the therapist. If they struggled, then may also struggle with the participant led sessions. Can this be checked? Or can the participant seek help from the therapist if require during the week? |
| Adaptations of the programme for different clinical settings could support the feasibility of the programme. | Expert 1: Yes [the duration of the programme is appropriate to achieve the aim of the programme], however 9-week program - individual may be difficult to implement in a clinical setting due to staffing in community / outpatients.  Expert 10: This may be difficult to staff if the intervention were rolled out across a large health service area staffed by community OTs [occupational therapists]. It may be easier to staff if it was rolled out in a residential setting with on-site therapists. |
| Traditional methods of repetitive task-orientated practice may compliment the app-based therapy. | Expert 7: Computer program does not relate to the reality for an older person in home environment. Better to stand with them in their own kitchen and repeat activity.  Expert 11: Actual practice of the task may be an important part of the program or contributor to outcomes? Do you think practice in the task should be considered as part of the program? |
| **Clarity** |  |
| The ‘drag and drop’ function is not an intuitive function for older people. | Expert 4: Instructions are clear and direct; however, some clients may find the drag and drop function difficult/unfamiliar.  Expert 5: More visual prompts [required] for tasks instructions / 'drop and drag' activities. |
| Improvements to the visual design and visual prompts to enhance clarity and improve the user experience. | Expert 2: Can consider adding the word "next" to the red box with arrow.  Expert 5: Consider colour of font i.e. contrast etc… Larger font, reduce wording where possible.  Expert 10: I can imagine some participants having issues with seeing the photos as they are quite small. |
| Feedback and progress updates would be beneficial for older people and therapists. | Expert 5: Consider immediate feedback for all activities i.e. prompt to state that the patient had made an error / had an incorrect response. Noted had this feature for some activities but not others.  Expert 8: Is it possible to include a progress bar to show participants where they are up to? |
| **Relevancy** |  |
| A tailored and individualised approach with an expansion of IADL will further support the use of the program in occupational therapy services. | Expert 1: Can the program be individualised? Some participants may not do those tasks; therefore, you may need to goal set with pt. [patient] to work on relevant items. This may shorten time frame of program.  Expert 9: If it could be personalised and shorter self-administered sessions, I would be much more likely to recommend. |
| Concerns for older people with limited previous exposure to technology. | Expert 3: I think it may require more than one [therapist-led session]. Very much depends on pt. background & their familiarity with technology.  Expert 8: Yes, I think so [one therapist led session is sufficient], although it may be more difficult for people who have never used an iPad before. |
| The current version of E-MinD Life is not for everyone. | Expert 3: Many of my patients are from CALD [culturally and linguistic diverse] background with reduced technological skills therefore could be challenging.  Expert 5: Many Pts [patients] are unable to use an iPad despite prompting. Pts [patients] are unable to read or have hearing impairments.  Expert 6: Older people with cognitive impairment may have difficulties using this type of technology esp. pts [especially patients] with visual impairment. |
